# A Multimodal Intention Detection Sensor Suite for Shared Autonomy of Upper-Limb Robotic Prostheses

**DOI:** 10.3390/s20216097

**Published:** 2020-10-27

**Authors:** Marcus Gardner, C. Sebastian Mancero Castillo, Samuel Wilson, Dario Farina, Etienne Burdet, Boo Cheong Khoo, S. Farokh Atashzar, Ravi Vaidyanathan

**Affiliations:** 1 Moonshine Inc., London W12 0LN, UK; marcus@moonshinegs.com; 2 Department of Mechanical Engineering, UK Dementia Research Institute Care-Research and Technology Centre (DRI-CRT) Imperial College London, London SW7 2AZ, UK; c.mancero-castillo16@imperial.ac.uk (C.S.M.C.); s.wilson14@imperial.ac.uk (S.W.); 3 Department of Bioengineering, Imperial College London, London SW7 2AZ, UK; d.farina@imperial.ac.uk (D.F.); e.burdet@imperial.ac.uk (E.B.); 4 Department of Mechanical Engineering, National University of Singapore, Singapore 119077, Singapore; mpekbc@nus.edu.sg; 5 Department of Electrical and Computer Engineering, New York University, New York, NY 11201, USA; 6 Department of Mechanical and Aerospace Engineering, New York University, New York, NY 11201, USA; 7 NYU WIRELESS, New York University, New York, NY 11201, USA

**Keywords:** shared autonomy, prosthetic technology, mechanomyography

## Abstract

Neurorobotic augmentation (e.g., robotic assist) is now in regular use to support individuals suffering from impaired motor functions. A major unresolved challenge, however, is the excessive cognitive load necessary for the human–machine interface (HMI). Grasp control remains one of the most challenging HMI tasks, demanding simultaneous, agile, and precise control of multiple degrees-of-freedom (DoFs) while following a specific timing pattern in the joint and human–robot task spaces. Most commercially available systems use either an indirect mode-switching configuration or a limited sequential control strategy, limiting activation to one DoF at a time. To address this challenge, we introduce a shared autonomy framework centred around a low-cost multi-modal sensor suite fusing: (a) mechanomyography (MMG) to estimate the intended muscle activation, (b) camera-based visual information for integrated autonomous object recognition, and (c) inertial measurement to enhance intention prediction based on the grasping trajectory. The complete system predicts user intent for grasp based on measured dynamical features during natural motions. A total of 84 motion features were extracted from the sensor suite, and tests were conducted on 10 able-bodied and 1 amputee participants for grasping common household objects with a robotic hand. Real-time grasp classification accuracy using visual and motion features obtained 100%, 82.5%, and 88.9% across all participants for detecting and executing grasping actions for a bottle, lid, and box, respectively. The proposed multimodal sensor suite is a novel approach for predicting different grasp strategies and automating task performance using a commercial upper-limb prosthetic device. The system also shows potential to improve the usability of modern neurorobotic systems due to the intuitive control design.

## 1. Introduction

The absence of the means to interact with the environment represents a steep barrier in quality of life for people with acquired or congenital upper limb deficiency. The vital role of the hand in basic activities of daily living (ADLs) is well-acknowledged [1]. However, matching the dexterity and complexity of the hand, which involves more than twenty degrees-of-freedom (DoFs), is still exceptionally challenging to capture artificially [2]. The development of advanced myoelectric prostheses in the last decades has led to innovative designs of multi-DoF control systems (e.g., [3,4]). However, a highly functional, robust, intuitive, and natural human–robot interface is yet to be developed. Currently, field operation of myoelectric prostheses relies on button activations, manual switching functions, and electromyography (EMG) input to iterate through different grasp modes. These devices commonly make use of model-free techniques that employ machine learning algorithms for the prediction of motor tasks, frequently requiring extensive calibration procedures to select and assign predefined activation patterns for driving the device [5]. For example, two EMG pulses may be associated with a “power grasp”, whereas a two-second cocontraction might be associated with a “tripod grasp”. Although this pattern-coding may allow for the generation of several functional postures, it is: (a) unintuitive, causing increased cognitive load; (b) results in slow, sequential, unnatural, and discrete posture control; (c) significantly increases the complexity of use of the prosthesis for the end user, requiring constant visual attention; and (d) requires excessive training [6,7]. Although high functionality and versatile design are imperative factors, they should not result in excessive complexity and a cumbersome control that degrades the intuitiveness and the ease of use of the device. This results in a trade-off between versatility and simplicity. A literature review on prosthetic user needs, conducted by Cordella et al., showed that the number one priority for users of upper limb myoelectric prostheses resides in functionality (i.e., the effort and ability of the user to perform different tasks based on the prosthesis capabilities), followed by prosthesis-related factors (i.e., comfort, appearance, etc.) [5]. The difficulty of use of the device and the lack of functionality have been reported to be major contributing factors for prosthesis rejection, which has been found to be around 25–35% for electric prosthetic users in a survey conducted by Biddiss and Chau [8] despite studies reporting the appearance of chronic pain due to changes in posture and overuse of the intact limb [9]. The mentioned control challenges have motivated significant research efforts on the development of advanced techniques that can process and decode biological signals using, for example, classification or pattern recognition schemes (e.g., [10,11]) to exploit the full discriminative power and data transfer rate of the signal for a higher level of control.

### 1.1. Existing Instrumentation Challenges

EMG is arguably the most studied technique to decode the biological code of motor intention [12] based on activities recorded from the stump muscles. Currently, most of the commercial EMG systems are based on dry electrodes due to the advantages in the setup procedure and skin preparation compared to wet contact electrodes [6]. However, there is an obstacle that affects every practical deployment of commercial EMG-based prosthetic systems. The main problem rests in the imperative need for consistency of the EMG signal, which is greatly affected by different factors particularly present in applications that require extended periods of device usage. These factors include the sensitivity of the signal to (a) sweating, (b) changes in arm posture, (c) fatigue, and (d) electrode misplacement/displacement [7], all of which cause significant degradation of the interpretation of the measured signal, making predicting computer algorithms, such as pattern recognition or classification, unreliable and ultimately resulting in the need of a constant and intensive calibration and maintenance procedure. These limitations become more evident in prostheses since the design of prosthetic sockets, along with the electrode housing method employed, have significant effects in the transduction of the EMG signal [13]. The EMG sensors used in these devices are affixed with some level of pressure within the socket to ensure a good level of conductivity; however, this leads to rapidly changing skin conditions due to the hindering of the natural mechanisms to cool down the body (a problem particularly present in amputee individuals who find it more difficult to lose body heat due to reduced skin area) [14]. Although latest advances on upper-extremity prostheses have allowed for more functional devices and solutions to minimize the extent of all the mentioned limitations [6,15], an economic solution for high-level prostheses that meet most of the user demands is still hard to obtain.

As an alternative approach, mechanomyography (MMG) has also been investigated in the literature [16]. It is used to record the mechanical responses of the muscle activation and motor unit recruitment during muscle contractions instead of the electrical activity of the motoneurons. MMG has been used in a number of studies involving the recording of muscle activity in both upper and lower limbs with highlighting potential for clinical applications [17]. In comparison to EMG, this mechanical modality (i.e., MMG) may have lower information transfer rate and discriminative power for classification of a high number of gestures. However, using MMG technology, some of the major practical difficulties related to the use of EMG can be addressed, such as electrode shifting and variability of the signal due to skin conditions [18]. In addition, the MMG signal can propagate through soft tissue, allowing the recording of the same at a distal location from the active muscle. Additionally, the frequency domain of the MMG signal, compared to EMG, has shown to provide with more relevant information of the contractile properties and fibre type composition of the muscles [19]. MMG has certain limitations, the most notable one being the sensitivity to the motion artefact; however, recent studies have proposed different methods to minimize the extent of this effect; for example, by continuously monitoring motion by the use of an inertial measurement unit (IMU) to differentiate between muscle activity and motion induced artefacts [20]. All the above mentioned advantages make MMG a good alternative modality for applications that require a lower information transfer rate while providing high robustness, less sensitivity to electrode-skin conditions, simple calibration and placement, and low costs for long-term in-home uses in an integrated system, such as the current study.

### 1.2. Shared Autonomy: A Potential Solution

In order to address the mentioned challenges, several studies have been initiated to decode motor intention and allow for conduction of multidirectional complex maneuvers while simplifying the control, minimizing computational cost, and maximizing the intuitiveness. For this purpose, the concept of shared autonomy has been introduced in the literature, referring to the detection and fusion of user intention with inputs from various sensory modules to enhance the control of the prosthetic device. The final goal is to improve the dexterity, functionality, and control, and to allow the user to utilize their natural posture control for interacting with the prosthesis in an intuitive manner outside the laboratory setting [3]. A very recent effort has been established in this direction to enhance and simplify the control of prosthetic devices [21]. This approach is inspired by the successful implementation of shared autonomy for adaptive cruise control of modern vehicles and autopilot systems. The main idea is to exploit the use of local contextual measurements to divide the task between the user and an intelligent control module embedded in the software architecture of the robotic limb [22]. The approach can reduce the cognitive load related to the low-level interaction while keeping the user in the loop for conducting the high-level intuitive control commands.

In the literature, a small body of work related to prosthetic control has been conducted to benefit from this concept. Došen et al. [23] proposed a method of using an autonomous controller to perform grasping activities. The controller first determines some properties of the object, such as size and shape, and based on that information it selects the appropriate grasp and aperture size. This work was extended by Marković et al. [24] using a stereo-vision system to select the grasp configuration and augmented reality glasses to provide artificial proprioceptive feedback. Further work by Marković et al. [25] focused on semiautonomous, simultaneous, and proportional control of a multi-DoF prosthesis. Using color and depth (RGB-D) vision system, the shape, size, and orientation of an object were estimated and integrated with kinematic data to select the correct grasp type. In a preliminary study [26], we utilized an eye-in-hand vision system for grasp configuration based on the estimated size of the target object. Tang et al. [27] extended this approach by applying a convolutional neural network (CNN) on data collected using a dynamic vision system to estimate object orientation and the needed grasp pattern. Similarly, a deep learning-based vision system was proposed by Ghazaei et al. [28], in which a CNN classifier was utilized to determine the grasp pattern without explicitly identifying the object dimensions. More recently, a technique was developed that combines deep learning and gaze information to classify objects and estimate its pose for grasping [29] and a novel method was proposed to categorize unfamiliar objects based on uncertain information derived from the features of the object [30].

The above-mentioned studies form the recently accelerated literature for using external contextual sensors, such as computer vision, to improve control of prosthetic devices through a shared autonomy technique. However, in practice, grasp selection and execution will not only depend on the morphology of the object, but also on the sensorimotor characteristics of the intended action. Therefore, it is necessary to accurately infer and track the user intention and fuse this information with other sensory modules and contextual information to properly direct and control a prosthetic device based on the decoded intended effort [31].

### 1.3. Contributions of Work

In this study, we propose a new approach, to be used outside the laboratory setting, for grasp intent prediction based on the integration of multiple wearable sensing technologies while considering practicality, ease-of-use, wearability, low implementation difficulty, low computational power, and low cost. This integration of sensing modalities allows us to transform the usability of the independent components into a system, which collaborates with the user. More specifically, we present a sensor suite, which combines (a) machine vision for object recognition, (b) custom IMUs for motion tracking, and (c) MMG for measuring voluntary muscle activations. This paper aims to show that the contextual information regarding the kinematics of motion together with object characteristics and mechanical responses of muscle activation (i.e., MMG) can be fused to decode the user intention when reaching for an object to successfully perform object grasping, thereby overcoming the limits imposed by currently available systems in the interest of increasing the user acceptance rate of prosthetic devices. In this paper, the real-time implementation of the multi-modal sensor suite and the control architecture are introduced and tested. A user study is conducted, which includes timed activity-based experiments with ten able-bodied participants and one transradial amputee.

In summary, new contributions of this work include the following:

- Introduction of a multimodal sensing system for intention detection and grasp selection using mechanomyography, computer vision, and inertial measurement units.
- Real-time implementation and evaluation of the multimodal system using low-complexity algorithms.
- Minimizing the calibration time due to the novel sensory fusion.
- User study evaluation, including timed activity-based experiments with ten able-bodied participants and one transradial amputee.

## 2. Methods

### 2.1. Human–Robot Interface Architecture

**Figure 1:**
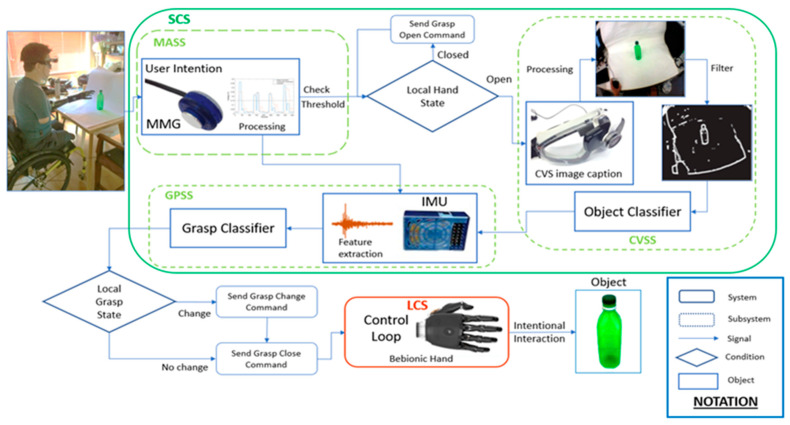
System overview of the heterogeneous sensor suite. The control architecture can be separated into a local control system (LCS) and a sensor control system (SCS). The muscle activation subsystem uses a mechanomyography (MMG) sensor to monitor the operator’s muscle response against an activation threshold. The pipeline consists of the computer vision subsystem (CVSS) and the grasp prediction subsystem (GPSS). The output of the SCS is sent to the LCS, where it is translated into motor commands to engage in intentional interaction.

The overall hierarchical design of the proposed system is depicted in Figure 1. The control architecture is divided into two major systems: the low-level local control system (LCS), responsible for the internal control of the prosthetic hand, and the high-level sensor-based control system (SCS), which fuses multi-modal information to generate the commands to drive the prosthetic hand. The SCS is divided into three major modules: muscle activation subsystem (MASS), computer vision subsystem (CVSS), and grasp prediction subsystem (GPSS).

#### 2.1.1. Muscle Activation Subsystem

This module is designed to monitor muscle activation using our state-of-the-art custom-made acoustic MMG sensor, introduced in our previous work [20]. One MMG sensor was sewn into a compression sleeve and connected to an IMU used for record upper-limb kinematics. The raw acoustic signal from the MMG sensor was filtered using a second-order band-pass Butterworth filter at a frequency range of 10–100 Hz. The filtered signal was then rectified and passed through a Hilbert transform to estimate the instantaneous envelope of the signal. The absolute value was then taken and compared against a pre-set threshold to detect intentional muscle activation. To prevent the system from mistakenly detecting the artefact as an activation signal, the system actively monitored the kinematics of the arm in real time to distinguish between muscle activity and motion induced artefacts, as described in [32]. In addition, two thresholding parameters were used, as described in [33]. The first threshold quantifies the energy of the MMG signal to detect intentional activation. The second threshold captured the energy of the gyroscopic information to discard the activation when the rotational acceleration of the arm surpassed a predefined value. The gyroscopic threshold was set during calibration of the IMU. The MMG threshold was set by recording 10 s worth of MMG data while the participant activated the appropriate muscle several times. No additional calibration was required. Once the activation threshold was reached and the motion conditions determined there was no arm movement, the control signal was passed on to the CVSS.

#### 2.1.2. Computer Vision Subsystem

The CVSS applies a simple and computationally inexpensive algorithm to process the images taken by a single low-cost Logitech C525 HD camera, which has a clip-on 180° fish-eye lens in an eye-to-hand configuration fixed to a pair of glasses worn by the user. The CVSS is designed based on basic processing elements to be easily implemented in most of the commercial integrated processing units with minimum need for memory and computational power. This subsystem initiates by taking a single snapshot of the operator’s point of view. Images are taken at a low pixel resolution of 640 × 480. Tilt and rotation angles are adjusted to centre the image with the user line of sight. The camera setup does not occlude the vision of the user. The captured image is enhanced using contrast correction, then converted to grey scale and subsequently processed for edge detection purposes. While more complex techniques are available [34,35], the computational intensity and challenges in implementing them into embedded code systems on board of the Bebionic hand dictated the implementation of leaner algorithms. In this paper, we utilized light image processing approaches to simplify the computation and maximize practicality and translation to the clinic. Due to this, we intentionally avoided using an advanced image processing module to evaluate whether basic algorithms can give us enough information to fuse the image-based features with mechanomyography for predicting the intention of the user. For this, in the image processing module, edge detection was performed by applying Sobel and Canny edge operators. Subsequently, the image was smoothed by a 5 × 5 Gaussian filter given by (1), with *σ* = 3 to merge nearby pixels to enclosed regions.

(1) $$G\left(x,y\right)=\frac{1}{2\pi \sigma^{2}}e^{-\frac{x^{2}+y^{2}}{2\sigma^{2}}}$$

A binary filter was applied to enhance the detected edges using a threshold. Image processing results in the detection of numerous blobs, which are filtered based on a number of properties to find the object of interest after several experiments to fine tune the parameters considering various objects and various angles taking into account the context of the task. This filter removes blobs with pixel density A, below A_min_ = 10 and above Amax = 5000, and height and width greater than H_cam_ = 480 and W_cam_ = 640, respectively. Blobs with a difference in Euclidean distance E_Dist_, from its centre of gravity to the centre of the image, above the distance threshold E_cen_ = 125, were removed. Since the camera was head-mounted, it was assumed that the subject’s gaze is in line with the centre of the camera; therefore, blobs with E_Dist_ > 125 were considered not to be within the subject’s visual interest. The remaining blobs were given a score B_Score_ according to Equation (2) and the highest scoring blob is identified as the object of interest.

(2) $$B_{Score}=\frac{A}{E_{Dist}}$$

**Figure 2:**
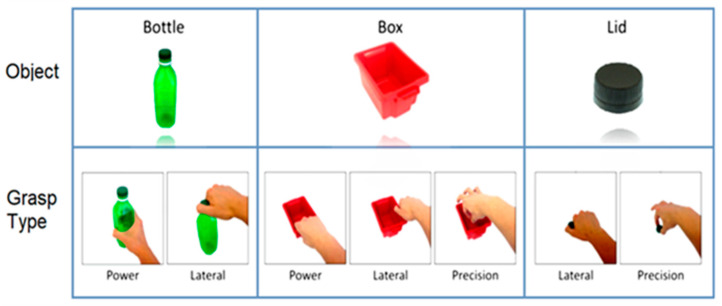
Three objects and seven grasp types used for the experiment. Twenty images are taken from various viewpoint angles for each object to construct the training dataset. The bottle was grasped using power and lateral grasps. The box was grasped using power, lateral, and precision grasps. The lid was grasped using lateral and precision grasps.

Properties from the resulting region of interest were calculated from its filled convex hull to be used as the feature space for object classification. The properties are listed in Table 1. During the training phase, each object template was generated by collecting its properties using 20 images taken at different viewpoints. During real-time object recognition, a K-nearest neighbour (KNN) classifier was used to identify the object of interest by comparing the extracted features to those collected in the corresponding object template. This classifier will be referred to as the object classifier (OC) throughout the rest of this paper. The detected object specified a set of possible grasp patterns unique to that object (see Figure 2), which were then used by the GPSS to determine the most suitable grasp.

#### 2.1.3. Grasp Prediction Subsystem

After the object of interest was detected by the CVSS, the corresponding set of object-specific grasp patterns were given to the GPSS. This subsystem had the role of data monitoring, data fusion, and command generation. As the participant generated an intention to reach for an object, the GPSS was initiated using the signal produced by the MASS. Thus, the GPSS started recording motion data using a pair of custom IMUs to estimate the kinematics of motion (position and orientation). The IMUs were affixed to the wrist and the biceps to capture kinematic features of the forearm and upper arm, respectively.

Orientation was estimated using a gradient descent algorithm developed by Madgwick et al. [36]. The gradient descent algorithm was computationally less demanding and can operate with lower sampling rates, enabling applications in low-cost, wearable IMU systems that are capable of running wirelessly over extended periods of time. This information was captured in the form of quaternions.

Displacement was measured with respect to the resting position (origin) and the object depth and angle locations, according to the workspace layout. During the reaching phase, kinematic information was collected and timestamped at a frequency of 1 KHz by the use of the IMU.

We initially focused on using distribution features of the quaternion displacement trajectory during different grasp patterns. Although this approach proved to be relatively successful, varying the position of the object reduced the classifier accuracy considerably due to the high variability in the arm’s movement trajectory, impeding the ability to discriminate between different grasps. As a result, individual vector components representing the change in displacement of a single point were analysed in this study by considering the X, Y, and Z quaternion components as an imaginary vector (the imaginary quaternion vector part) in 3-dimensional space with an origin at [0,0,0] (resting position). In this study, rather than only looking at the distribution features of the whole displacement profile, single point features were also considered and tracked. A total of 84 kinematics features were extracted from displacement, velocity, and acceleration.

**Figure 3:**
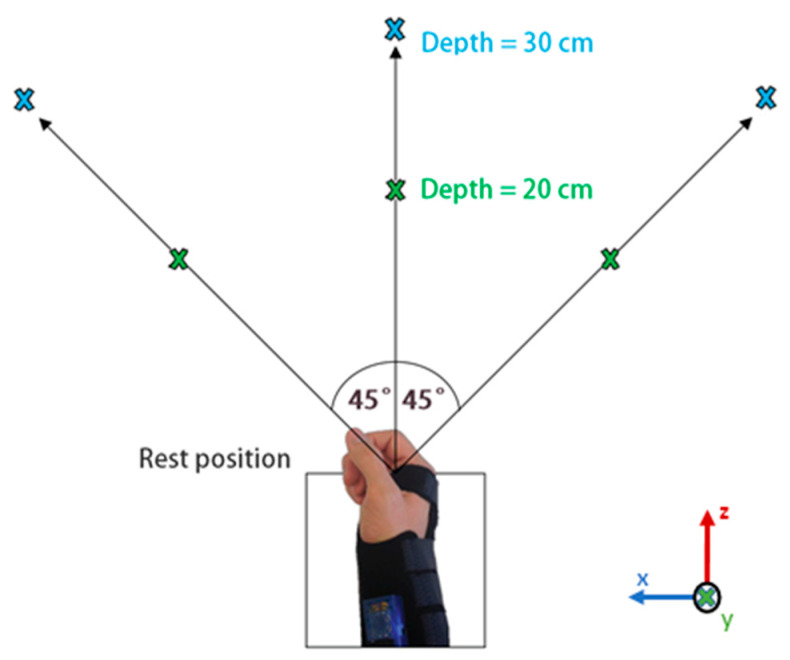
Object locations during data collection at −45°, 0°, and 45° with depths of 20 cm and 30 cm. The same locations are used at three elevation levels: ascending, descending, and table height.

A list of the 14 most informative types of kinematics features selected based on the scoring method described in the next section (Remark—Feature Selection for Grasp Classification), is shown in Table 2. The maximum value along the positive vector axis is denoted as Max(+) and the maximum value along the negative vector axis is denoted as Max(−). These 14 types of features were calculated for the six segment motions (mentioned below) resulting in the 84 kinematic features. The segment motions were related to the forearm and upper arm Cartesian space in X, Y, and Z directions as illustrated in Figure 3, represented by the terms FAx, FAy, and FAz, for the forearm and UAx, UAy, and UAz, for the upper arm (e.g., PoG:Disp-FAx refers to the final displacement of the forearm at the point of grasp in the x direction).

The object detected by the CVSS determines the set of possible grasp patters to be established by the GPSS. After a 3-s window of data recording during reach, inherent physiological features (estimated from the IMU data) were extracted and compared against a pre-existing subject- and object-specific grasp template to classify the intended grasp using a second KNN classifier, referred as the grasp classifier (GC) in the rest of this paper. Once the intended grasp was identified based on the augmented feature space (fusion of muscle activity, contextual information, and kinematic data), the appropriate output command corresponding to the selected grasp pattern was generated and sent to the LCS for the execution.

Remark—Feature Selection for Grasp Classification: As mentioned, the grasp classifier works based on the fusion of multi-modal information including the kinematics of motion during the reaching phase. However, many trajectories can be chosen by an individual when reaching to grasp an object. These trajectories can cause variations in the forearm and upper arm orientation, velocity, and acceleration. Furthermore, the reaching paths may vary depending on the location of the object, thus increasing the variability and redundancy in the feature space. In order to augment the input data, several different reaching trajectories and object locations were considered in the training phase. For this, the input space (which includes MMG data, contextual vision-based information, and kinematics of motion) was recorded under multiple variations of (a) elevation/declination, (b) angular rotation, and (c) depth relative to the participant. Subsequently, in order to reduce the computational cost, the minimum number of required features for classification was calculated using a sequential feature selection method. This process involves the creation of candidate feature subsets by sequentially adding each feature in turn and performing leave-one-out cross-validation using a criterion defined by the KNN classifier (which uses a Euclidean distance metric to measure feature performance). The criterion returned by the classifier was summed and divided by the number of observations, which were used to evaluate each candidate feature subset by adding features one by one based on the minimization of the mean criterion value, the error rate. This error rate was estimated as the average error of all the folded partitions according to (3), where 𝐸 is the true error rate, K = N is the number of folds (with 𝑁 as the total number of samples), and 𝐸𝑖 is the error rate for fold 𝑖.

(3) $$E=\frac{1}{N}\overset{N}{\underset{i=1}{\sum }}E_{i}$$

The top 10 features were selected, ordered based on ascending error rate, and scored according to the order they were selected through forward sequential feature selection. Namely, each feature selected was issued a score 𝑆 given by (4), where 𝑅 is the position of the feature in the ordered selection based on the error rate, and 𝑆_𝐶_ (equal to 1) was the maximum initial score. The mean scores were then calculated across all participants to identify the most important features during the grasp.

(4) $$S=S_{C}-0.1\left(R-1\right)$$

#### 2.1.4. Control Flow

The decision flow of the system for when the user intends to interact with the object of interest is the following: the process initiates with the subject focusing the object on the centre of the camera’s point of view. The user activates the forearm muscles generating a signal activation that is captured by the MMG sensor. Gyroscopic information from the IMU is acquired simultaneously and compared against a threshold value in order to differentiate the activations signal between motion artefact and intentional activation. If intentional activation is detected, the system sends a command to the Bebionic hand to open grasp (hand open). Subsequently, the CVSS is used to take a snapshot of the camera’s range of view. Image processing is then performed in order to detect the region of interest to classify the object based on its morphology and according to the template using the object classifier. Once the object is identified, the participant is prompted by an audio cue to reach towards the object within a period of 3 s, with the intention to perform a specific grasp action. During the reaching phase, kinematic information is recorded from the forearm and upper arm by the use of the IMU sensors. After the three seconds of data recording, grasp features are extracted and compared against the user-specific grasp template for the object class determined previously, predicting the desired grasp pattern according to the grasp classifier. Once the correct grasp pattern has been selected, a grasp close command is sent to the Bebionic hand in order to close the hand around the object. The system is then reset to capture MMG data again with the next intentional activation resulting on an open grasp command in order to release the object.

#### 2.1.5. The Low-Level Local Control System

In this work, the Bebionic V2 Hand was utilized. This device is traditionally controlled by the use of button activations, EMG signal inputs, and manual operation. For the purpose of this study, the entire control system of the hand was replaced by the use of the present multi-modal approach. In the LCS, the EMG signals previously driving the prosthetic hand were replaced with commands, which convert the trigger signals made by the GPSS (produced based on the fusion of MMG, computer vision, and kinematics) into voltage commands to drive the Bebionic Hand. Using the proposed framework, the autonomy of the position space of the hand was achieved in order to enhance the position control of the device. Factory settings with default operation of force and speed were used. The hand was configured to allow for 8 grasp patterns to be controlled using external software (i.e., LCS).

### 2.2. Demographic Data

Ten abled-bodied right-handed individuals (9 males and 1 female) and one transradial amputee (male), between the ages of 22 and 37 years old, participated in two experiments that took place on different days. All subjects gave their informed consent before taking part in the study. All experiments were approved by the Imperial College Research Ethics Committee (ICREC reference: 15IC3068).

### 2.3. Experimental Protocol

The experimental protocol for this study was divided into two parts. The first phase of the experiment consisted of the collection of data for training the two interconnected classifiers (OC and GC). The second phase consisted of the real-time performance evaluation of the proposed multi-modal sensor suite during pick-and-place tasks.

#### 2.3.1. Phase 1—Data Collection Protocol

During data collection, three objects (bottle, lid, and box) were chosen to carry out the selected grasp patterns as shown in Figure 2. The bottle was assigned with two grasp patterns: a power grasp for transporting, and a lateral grasp for unscrewing the lid. The lid was assigned two grasp patterns: a lateral grasp for screwing on the lid, and a precision open grasp for transporting the lid. The third object was assigned with three different grasps: a power grasp for transporting the empty box by grasping it from the front; a lateral grasp for transporting the filled box by grasping its side; and a precision open grasp for picking up small objects inside the box. A total of 7 classes for grasp patterns were considered.

The experimental layout is illustrated in Figure 3. Each object was tested consecutively at each one of the 18 possible locations. Data was recorded with the object at angular positions of −45°, 0°, and +45°, and depths of 20 cm and 30 cm for each of the three following positions of the user:

- Table height: object placed on the table while user seats.
- Ascending: object raised 15 cm on table while user seats.
- Descending: object placed on the table while user stands.

The amputee took part in the data collection of objects at table height and ascending positions only. To ensure a systematic evaluation of the tasks for every participant, a 3-s time window was given to the user to reach and grasp the object of interest from the resting position once the initiation of the experiment was prompted by the software. The amputee was not required to grasp the object with the prosthesis but only to intend, move, and orientate the hand to the grasp point location. The time-window duration was chosen to give the user sufficient time to perform the task. The present framework allows for the flexibility of duration (shorter or longer) of this time-window. Once the object is grasped, the user kept the hand in the grasp position for the remainder of the 3-s interval until prompted to return to the rest position. A 2.5-s time interval was given for returning to the rest position. This process was repeated for five trials at each object location for each grasp pattern, taking approximately one hour in total per each session. The order of tasks was randomized.

For able-bodied participants, the user’s dominant hand was positioned at rest, with the centre of the palm approximately 10 cm away from the edge of the table. The chair was adjusted so that the user’s elbow was bent at approximately 90°. When standing, the arm was pointing downwards, approximately in line with the centre of the rest position. The amputee was seated on his own wheelchair. The upper arm sensor sleeve was fitted further up on the arm so that the residual limb had space to fit into the forearm prosthesis socket. The forearm socket was fitted to the Bebionic V2 hand using a custom-made adapter and the forearm sensor sleeve was fitted over it.

#### 2.3.2. Phase 2—Real-Time Task Conduction and Assessment Protocol for Classification Performance

On the second phase, the following protocol was practiced:

(a) Tests on able-bodied users: the compression sleeve, which was equipped with the IMU and MMG sensors, was pulled over the participant’s right arm (dominant arm of all participants of this study). An IMU was positioned on the biceps and the MMG was positioned on the forearm over the extensor digitorum. The edge of the compression sleeve was pulled over the shirt sleeve and additional straps were used to ensure a tight fit. The Bebionic hand was connected to the adapter, with the wrist splint containing a second IMU. The participants held the hand between the thumb and fingers. Velcro straps were used to keep the hand in place, preventing flexion or extension of the wrist.

(b) Test on amputee: the compression sleeve was fitted higher up on the upper arm. The MMG sensor was positioned on the biceps instead of the forearm. The vision system along with an audio headset (to prompt the user action) was placed on the participant’s head. The tilt and angular position of the glasses were adjusted to be centred with the line of sight of the user.

The workspace consisted of a table divided into three sections, each of 15 cm × 25 cm. For this phase of the experiment, the rest position was 25 cm in line with the central cordon, and the objects were kept at table height. The objects and grasp patterns were tested in the following order:

- Bottle (power and lateral);
- Lid (lateral and precision);
- Box (power, lateral, and precision).

**Figure 4:**
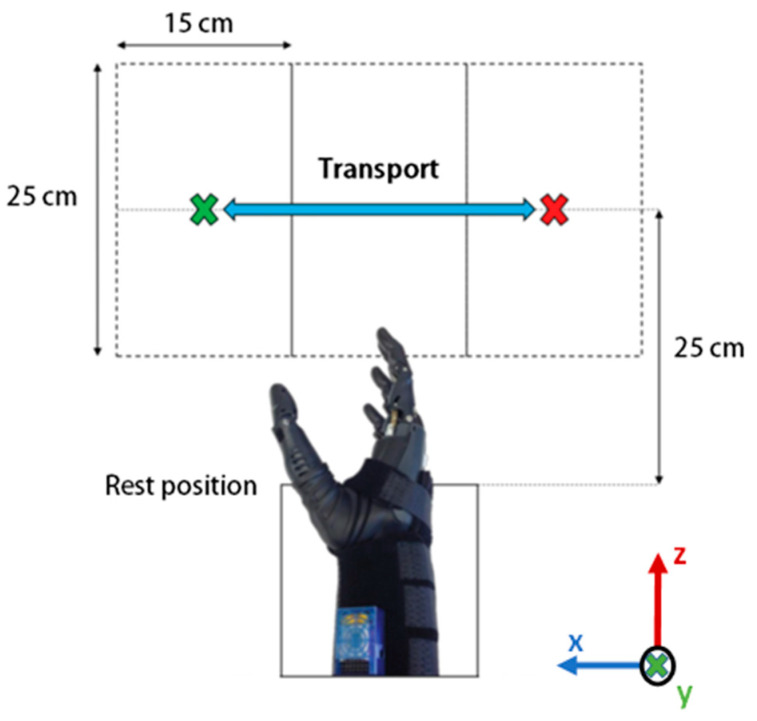
Top view of the classification performance assessment workspace. The object is placed in the centre of the left cordon and is transported towards the right cordon using a specific grasp pattern, and vice versa. The hand starts and ends at the rest position.

The first object (bottle) was positioned in the centre of the left cordon and the hand was placed in the rest position. When prompted, the participant generated the intention and moved towards the object to make the first grasp. This muscle contraction was recorded by the MMG, generating the initiation of the system. The integrated camera was continuously taking snapshots during task execution. Once found in the viewpoint, the OC determined the object of interest. Morphological information was then fused with MMG and kinematic data. The generated augmented input space was then processed by the GC to predict the most appropriate grasp based on the multi-modal measurements. As soon as the intended grasp was predicted the control command was generated and communicated to the control loop of the Bebionic hand to produce the intended grasp. Once grasped, the participant transported the object from the left to the right cordon, released the object, and returned to the rest position. An illustration of the different states is shown in Figure 4. For this study, the experimental protocol was designed considering a workspace proximal to the torso to simulate the interaction of the user with an environment similar to those of daily tasks. The working area is primarily defined by the range of vision of the CVSS and the position where the object lays, which in this case is limited to the torso area. However, the system is flexible enough to allow for the expansion of the workspace to locations other than those proximal to the user.

**Figure 5:**
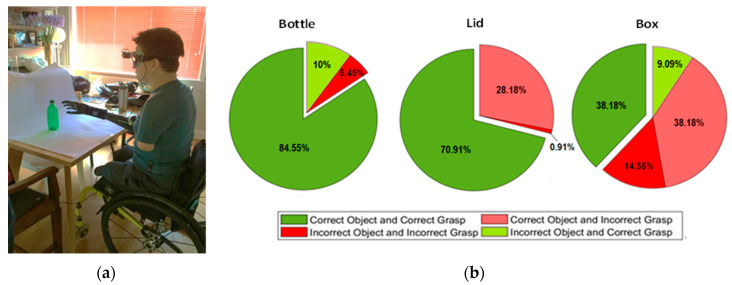
(**a**) The amputee is carrying out the classification performance assessment on a bottle using a power grasp. (**b**) Pie charts displaying the average classification outcomes for each object across both able-bodied and amputee participants. In total, there were seven classes of grasp pattern. The outcome is said to be true-grasp-true-class if the object and grasp were classified correctly and true-grasp-false-class if the grasp was correct, but the object was classified incorrectly. A false-grasp-false-class response occurred when both object and grasp were classified incorrectly and a false-grasp-true-class occurred if the correct object was identified correctly, but the wrong grasp was chosen.

In this phase, five trials were performed for each grasp pattern. To evaluate the performance, the object classification rate and grasp classification rate were calculated for each grasp. Figure 5 shows the amputee performing the second phase of the experiment (test phase). As can be seen, using the proposed shared autonomy technique and the multi-modal sensor suite, the amputee was able to achieve high performance during the test phase, particularly for correctly grasping the objects. A video of the system interacting with two objects in a pretrial test is found at: https://www.youtube.com/watch?v=KVHbDsGk1×8.

## 3. Results

### 3.1. Kinematic Features

The computation of the distribution of discrimination rates for the kinematic features showed that displacement features were the strongest on average, and acceleration features provided the lowest discrimination overall. The overall distribution of displacement, velocity, and acceleration features across all objects and participants gave an average of 60.75%, 33.39%, and 5.86%, respectively. Looking at the forearm and upper arm split, the overall forearm and upper arm features resulted in 56.16% and 43.84%, respectively.

**Figure 6:**
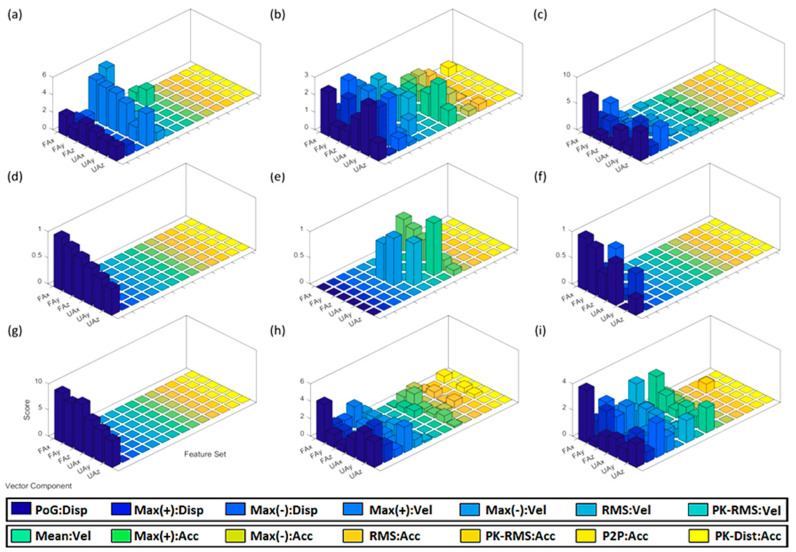
Cumulative kinematic feature scores of able-bodied participants grasping a (**a**) bottle, (**b**) lid, and (**c**) box across all object locations. Feature scores of amputee individual grasping a (**d**) bottle, (**e**) lid, and (**f**) box at table height and ascent. Cumulative modified feature scores of able-bodied participants grasping a (**g**) bottle, (**h**) lid, and (**i**) box at table height and ascent.

Figure 6 shows cumulative kinematic feature scores for able-bodied participants (Figure 6a–c), amputee participant (Figure 6d–f), and cumulative kinematic feature scores for all participant (Figure 6g–i). It shows the introduced 84 kinematic features for displacement, velocity, and acceleration of different motion segments in the three Cartesian directions.

Feature scores of able-bodied individuals for grasping the bottle are shown in Figure 6a. Max(−):Vel-FAx shows the highest score with a value of 5.5, based on the scoring method given by (4). Feature scores for the amputee grasping the bottle are shown in Figure 6d. PoG:Disp-FAx showed the highest score of 0.85. Feature scores for able-bodied individuals grasping the lid are shown in Figure 6b. PoG:Disp-UAy and Max(−):Disp-UAy showed the highest scores, with values of 2.9 and 2.7, respectively. Feature scores for the amputee grasping the lid are shown in Figure 6e. Mean:Vel-UAy had the highest score, resulting in a value of 0.9. Feature scores for able-bodied participants grasping the box are shown in Figure 6c. PoG:Fax showed the highest score of 7.2. Feature scores for the amputee grasping the box are shown in Figure 6f.

The proposed technique was found to behave differently between grasps made at the descending position and grasps made at the other two positions (i.e., table height, and ascending). To further analyse the performance of the technique with respect to object location, data corresponding to able-bodied participants at descending position was excluded for the rest of the analysis. This is to better compare the results between able-bodied participants and the amputee, who only took part in the test for objects at table height and ascending positions. The resulting feature scores are shown in Figure 6g–i, for the bottle, lid, and box, respectively. These scores were compared to those of the amputee as they cover the same object locations.

**Figure 7:**
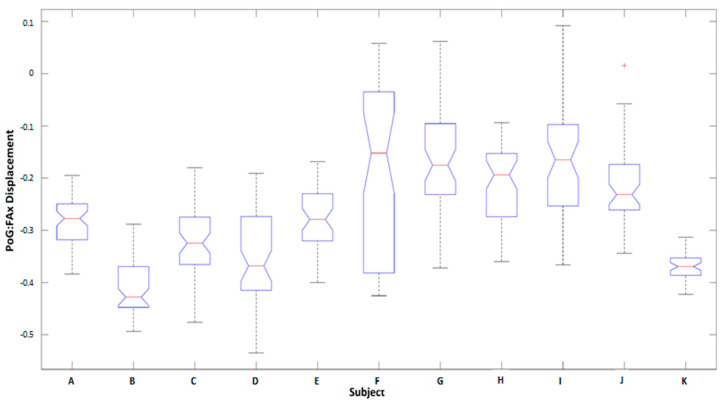
Comparison of individual subject PoG:Disp-FAx distributions for lateral grasps made on the bottle at table height and ascending positions. The median and variance of the feature are considerably varied between subjects. Participant K is the amputee.

Analysis of intrasubject variation of kinematic features (Figure 7) shows the subject-based distributions of PoG:Disp-FAx (the most informative feature, as discussed in Section 4.1.2) for lateral grasps made on the bottle across all subjects for all trials, taken at the table height and the ascending position (for comparison between able-bodied and amputee participants). As shown in the figure, the median, and the variance values of the features were significantly different between subjects. The amputee (subject K in Figure 7) shows a median magnitude of −0.37 and a very low standard deviation. The resulting *p*-values calculated based on a Kruskal–Wallis test for comparisons between two pairs of distributions given in Figure 7 were less than 0.001. Overall, the average *p*-values from the Kruskal–Wallis test across all features for all grasps and object classes rejected the null hypothesis at 𝛼 < 1% significance. Consequently, identifying the best features across subjects would not be feasible as arm motion features were significantly different between participants, showing the need for subject-specific templates.

### 3.2. Classification Performance Assessment Evaluation

**Figure 8:**
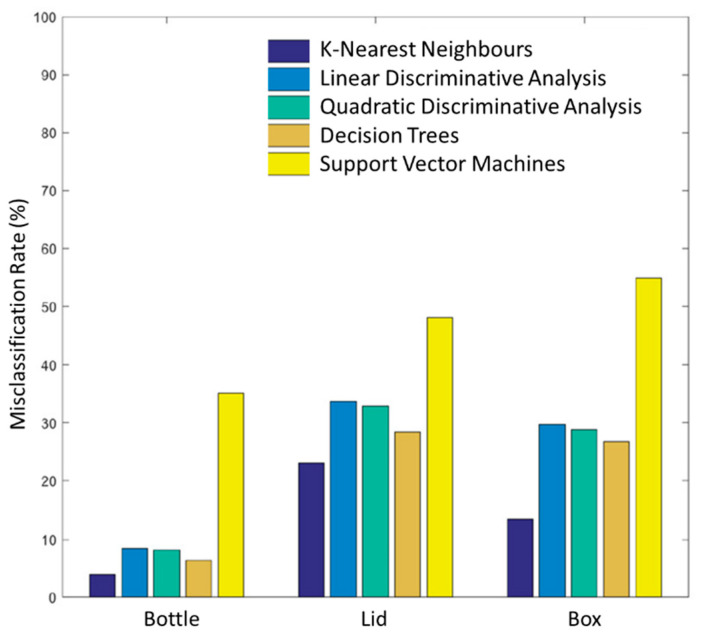
Comparison of mean misclassification rates for 10 able-bodied subjects for bottle, lid, and box objects across for all object locations. The K-nearest neighbour (KNN) classifier performed the best across all objects, with a mean misclassification rate of 3.83%, 23.03%, and 13.59% at all object locations for the bottle, lid, and box respectively, while SVM performed the worst.

The mean misclassification rate across all 10 healthy subjects is given in Figure 8, a comparison between five different classifiers was carried out including KNN, linear discriminative analysis (LDA), quadratic discriminative analysis (QDA), decision tree (DT), and support vector machine (SVM) classifiers for grasps made across all object locations. Overall, the KNN classifier performed the best across all objects, while SVM performed the worst. Similar classifier performance was found for the amputee subject.

The KNN grasp classifier shows an average grasp classification rate for bottle, lid, and box of 96.17%, 76.97%, and 86.41%, respectively for all object positions for able-bodied and amputee participants. Excluding the data at the descending position for able-bodied participants (for comparison between able-bodied and the amputee) resulted in a classification accuracy for the bottle, lid, and box of 100%, 81.65%, and 88.49%, respectively. For the amputee, the classification accuracy for bottle, lid, and box was significantly higher, yielding 100%, 96.64%, and 95.53%, respectively. Combining the results across both demographics yielded an average classification accuracy rate of 100%, 82.46%, and 88.88% for bottle, lid, and box, respectively.

The overall success rate of grasp prediction for the bottle across all participants was 98% and 100%, respectively. Grasp prediction of the lid object was 71% and 80% for able-bodied and amputee participants, respectively. Grasp prediction for the box resulted in a classification accuracy of 71.6% and 66.7% for able-bodied and amputee participants, respectively.

## 4. Discussion

### 4.1. Analysis of Kinematic Features

#### 4.1.1. Observations Related to All Object Positions

Observations related to bottle object: As can be seen in Figure 6a, Max(−):Vel-FAx had the highest score (average of 5.5). However, results showed that Max(+) velocity features performed the best across all the other forearm and upper arm vector components. Velocity features performed the best among all three motion profiles. Feature scores for the amputee depicted in Figure 6d show that PoG:Disp-FAx had the highest score (average of 0.85).

Observations related to lid object: As shown in Figure 6b, the two strongest performing features were PoG:Disp-UAy and Max(−):Disp-UAy. Displacement features outperformed features from velocity and acceleration profiles. Feature scores for the amputee, shown in Figure 6e, show that upper-arm velocity features appeared to provide the strongest separation between lateral and precision grasps.

Observations related to box object: As can be seen in Figure 6c, the displacement profile had the strongest scores, the highest being 7.2 for PoG:Fax. Feature scores for the amputee grasping the box shown in Figure 6f denote how displacement features performed the best. PoG:FAx provided the highest discrimination between lateral, power, and precision grasps. Feature scores showed similar behaviour to those of able-bodied individuals where displacement features were also dominant.

#### 4.1.2. Observations Related to Table-Height/Ascending Positions

Observations related to bottle object: Comparing feature scores from able-bodied and amputee individuals for the bottle (Figure 6d,g) shows very similar behaviour. As can be seen in the figures, PoG:Disp features provide the largest discrimination capability between lateral and power grasps, with forearm features being the most informative.

Observations related to the lid object: The most prominent features for grasping the lid were displacement features, which showed stronger discriminative capability for able-bodied individuals. Velocity features also played a considerable role in the classification of data collected from the amputee.

Observations related to box object: Feature scores for the box show that for able-bodied individuals and the amputee the displacement features were the most prominent. However, velocity features also played an important role in grasp discrimination, particularly for able-bodied participants.

#### 4.1.3. Comparison: Displacement, Velocity, and Acceleration

Acceleration features provided the lowest discrimination power overall, whereas displacement features were the strongest on average. In the case of the amputee, for two out of the three objects, displacement features encoded almost all kinematic information used for discrimination. Velocity features still had a reasonable impact on discrimination. In summary, displacement features performed the best, compared to velocity and acceleration features and forearm features performed better than those from the upper arm. These results suggest that upper arm motion is as important as forearm motion when determining grasp.

### 4.2. Classification Performance Assessment Evaluation

The higher performance of the classifier for the amputee subject compared to able-bodied participants may be due to less possible variations (and thus less standard deviation in the feature space, as shown in Figure 7) for task performance due to the absence of the biological limb. This increased performance supported the feasibility and usability of the proposed multimodal sensor suite to be used as an embedded component for bionic hands.

Observations related to bottle object: The overall success rate of grasp prediction for the bottle across all participants was very high compared to the other two objects. It should be noted that, following the instances of correct classification of the object, based on image classification as bottle, both power and lateral grasps for both demographics resulted in an accuracy of 100% for grasp prediction. This suggests that all misclassifications occurred during vision-based object identification.

Observations related to lid object: Grasp prediction of the lid was reasonably successful. For able-bodied individuals, the grasp classifier found the precision grasps easier to classify than the lateral grasps, resulting in 83.7% against a 60% success rate. Conversely, for the amputee participant, the classifier showed the classification accuracy of 100% for the lateral grasp whereas it only showed 40% for the precision grasp.

Observations related to the box object: On average, the proposed technique found it more difficult to select the grasp for the box. However, the success rate was approximately an even split between true-grasp-true-class and true-grasp-false-class outcomes. A large value of true-grasp-false-class classifications was observed as a result of the power and the lateral grasp being misclassified as the other.

To summarize, the proposed sensor suite showed high performance for (a) identifying the correct object and (b) predicting the intended grasp when interacting with the bottle and lid objects. For these cases, classification success rates of 84.55% and 70.91% were achieved, respectively. However, for the box object, 38.18% success rate was obtained for correctly detecting the object and assigning the correct grasp pattern. A performance of 76.36% was achieved for the prediction of the grasp pattern for the box object. These results indicate that in some trials for which the box was misclassified based only on the image data, the proposed multi-modal scheme technique was still able to correctly assign a grasp pattern.

It should be mentioned that certain grasp features vary when facing different kinematic conditions. For example, for a user whose main grasp template features rely on velocity or acceleration profiles, it would be more difficult to classify the grasp correctly when rushing to complete a timed task. Therefore, in terms of adaptability and generalizability for agile and dynamic movements, displacement features are desirable as they are less vulnerable to changes due to external factors. This highlights the importance of enriching the template of data by adding faster and highly accelerated motions.

Remark—limitations and future perspectives: In this study, we proposed the feasibility of use of a multi-modal sensing unit (i.e., vision, MMG, and kinematic modalities) using a common classification algorithm (i.e., KNN classifier) to detect user intent and classify grasp patterns to interact with different objects. It should be mentioned that the objects used have been selected as a sample representation of common household objects, considering that the grasp patterns required for their manipulation are comparable. The use of MMG activity as an alternative to EMG was considered in this study as it provides a more robust and reliable tool for unstructured environments. It should be noted that MMG has some level of sensitivity to motion artefacts, skinfold thickness and the amount of contact pressure applied on the sensor [16]. In order to address these limitations, this paper proposed a hybrid solution, which fuses information from human body and semantic information collected by an autonomous agent.

The computational load benefits discussed in this paper referred to the computational requirements of using conventional algorithms as opposed to more specialized techniques, which often demand high computational power. The present study did not claim to reduce the computational load by the use of the multi-modal sensing unit but instead shows the feasibility of the proposed framework for prosthetic control using a commonly utilized algorithm for classification of objects and image processing, which require low computational power. A comparison of the results with other classification algorithms and a comparison with other myographic modalities for prosthetic control is out of the scope of this paper. Future endeavours of this work include the use of a dual EMG-MMG modality, a wearable hand-held vision system embedded in the prosthetic device, and the use of shallow neural network for training of the model with a wider variety of different shaped and sized objects placed at different locations and perspectives to further tackle the challenges encountered outside the laboratory setting. It should be noted that the present study does not claim to overcome the performance of existing EMG-based systems but instead it proposes an integrated multi-modal sensing unit to replace and improve the control system of the Bebionic hand based on the concept of shared autonomy.

## 5. Conclusions

Most of the clinically available hand prostheses still rely on simple proportional binary control driven by button activations, manual mode switching operations, and EMG inputs, resulting in an unintuitive mediated process, which requires a high cognitive load on the user. This study reports the development of an integrated wearable multimodal sensor suite for grasp prediction using a shared autonomy framework, which combines information from machine vision, custom-made IMUs, and MMG sensors. In this study, a simplified image processing technique was used in order to maximize the practicality for in-home uses when a heavy but more expensive stand-alone processor is not viable, and when the environment is typically structured. Due to the modular design of the proposed system, the image processing method can be replaced by a more complex algorithm if needed. As mentioned in the literature, applications of the concept of shared autonomy, such as the current study, may have a positive influence in the cognitive load and muscle fatigue demand due to the dynamic of shared tasks. The objective evaluation of these two parameters is part of the future work of this paper. Similarly, in order to evaluate the proposed framework under more practical and challenging environments, an extended investigation including a higher number of participants and a comparable number of amputees is guaranteed.

By the use of the unique multimodal fusion of the proposed system, we showed that the user was able to utilize the system after a very simple and fast calibration process. We would like to highlight that the MMG calibration was set by recording only 10 s worth of MMG data. This calibration period shows great potential when compared to other conventional myographic methods, which are very extensive and often require multiple tuning sessions to set the correct activation of the prosthetic device. We also highlight that our MMG sensor has demonstrated itself capable of long-term (5+ hours) recording in the field for lower limb prosthetics [17] and for upper-limb stroke rehabilitation [37,38] and can be further enhanced by increasing the information context of it through the administration of normal force [39]. We concluded that the proposed multi-modal sensor suite is a novel approach for prediction of the intended grasp strategy and automation of task performance using upper-limb prosthetics. The ability to apply an intuitive control command to the bionic hand without the need of additional and potentially fatiguing gestures could vastly improve the usability of modern neurorobotic systems for prolonged periods of time and is a step towards an intelligent bioinspired mechatronic design. The sensing system has been patented [40] as a basis for commercial translation.

## Figures and Tables

**Table 1:** Object properties.

Property	Description
Perimeter	Number of pixels around the edge of the blob.
Area	Number of pixels within the entire blob.
Major-minor Ratio	Ratio of the blob’s major axis to its minor axis.
Eccentricity	Parameter denoting non-circularity of the blob.
Area-Axis Ratio	Ratio of the blob’s area to the bounding box.
Area-Ellipse Ratio	Ratio of the blob’s area to the bounding ellipse.

**Table 2:** Kinematic feature space.

Property	Description
PoG:Disp	Final displacement of the arm at the point of grasp (PoG).
Max(+):Disp	Maximum displacement during grasp in the + direction.
Max(−):Disp	Maximum displacement during grasp in the − direction.
Max(+):Vel	Maximum velocity along the positive vector axis.
Max(−):Vel	Maximum velocity along the negative vector axis.
RMS:Vel	Root-mean-square velocity.
PK-RMS:Vel	Ratio of the largest absolute velocity value to the RMS.
Mean:Vel	Mean average velocity.
Max(+):Acc	Maximum acceleration rates.
Max(−):Acc	Maximum deceleration rates.
RMS:Acc	Root-mean-square acceleration.
PK-RMS:Acc	Ratio of the largest absolute acceleration value to the RMS.
P2P:Acc	Maximum-to-minimum difference.
PK-Dist:Acc	Time distance between maximum and minimum peaks.
